# Supplementary material for: The Impact of the COVID-19 Pandemic on the Food Security of UK Adults Aged 20–65 Years (COVID-19 Food Security and Dietary Assessment Study)
Source: Nutrients. 2022 Nov 29;14(23):5078. doi: 10.3390/nu14235078 (PMC9736021; doi:10.3390/nu14235078)
Supplement: Supplementary file 1 [file nutrients-14-05078-s001.zip › nutrients-2019605-supplementary.pdf]

Supplementary Table S1. Schofield equations for estimating BMR (kcal/d) from weight (kg)

	BMR (kcal/d)	
Age (years)	Males	Females
10-17	17.7 x Wt + 658.2	13.4 x Wt + 692.6
18-29	15.0 x Wt + 692.1	14.8 x Wt + 486.6
30-59	11.5 x Wt + 873.0	8.1 x Wt +845.6
60+	11.7 x Wt + 587.7	9.1 x Wt + 658.4

WT = weight

Table adapted from PILOT-PANEU project

Supplementary Table S2. Physical Activity Level (PAL) values for category of physical activity (age dependent) and the corresponding lower and upper cut off values

Age group (years)	Category of Physical Activity	PAL	Lower Cut-off	Upper cut-off
18-69	Low	1.4	0.872	2.249
	Moderate	1.6	0.996	2.570
	Vigorous	1.8	1.120	2.892
70-74	Low	1.4	0.872	2.249
	Moderate	1.6	0.996	2.570

PAL = Physical Activity Level

Table adapted from PILOT-PANEU project
